# Utilization of electronic health records and associated factors among nurses in a faith-based teaching hospital, Ilishan, Nigeria

**DOI:** 10.1093/jamiaopen/ooad059

**Published:** 2023-08-04

**Authors:** Love B Ayamolowo, Omolola O Irinoye, Abayomi S Olaniyan

**Affiliations:** Department of Nursing Science, Obafemi Awolowo University, Osun State, Nigeria; Department of Nursing Science, Obafemi Awolowo University, Osun State, Nigeria; Babcock University Teaching Hospital, Ogun State, Nigeria

**Keywords:** electronic health records, utilization, factors, nurses, health informatics

## Abstract

**Introduction:**

It has been documented that nurses’ use of electronic health records (EHRs) impacts clients’ health outcomes positively. Some health facilities, primarily privately owned institutions, introduced EHRs for optimal healthcare. Evidence of such and associated factors among nurses must be documented to improve utilization and quality.

**Objective:**

The study assessed the utilization of EHRs and associated factors among nurses in a faith-based teaching hospital.

**Materials and Methods:**

This sequential explanatory mixed-methods study involved a sample of all 240 nurses from a teaching hospital where EHRs have been introduced. Quantitative data through semistructured questionnaires were collected and analyzed using Chi-square and logistic regression. Qualitative data were collected from 10 purposively selected nurses using an in-depth interview guide and analyzed through content analysis.

**Results:**

The majority of participants reported availability of EHR computer software (62.8%), internet facility (84.2%), and desktops (76.3%), but EHR was poorly utilized (27.3%). Factors significantly associated were nurses who were females [OR (odds ratio) = 1.5, 95% CI (confidence interval), 0.21–11.24], BNSc degrees holders [OR = 4.3; 95% CI, 1.06–17.43]; had computer EHR software [OR = 7.4, 95% CI, 0.83–3.81], and sponsored EHR training [OR = 2.10; 95% CI, 0.24–18.6]. Noncapturing of nursing tasks and nursing standardized language by EHR software, lack of institutional enforcement on EHR use, and absence of clear EHR policies were the main identified themes for the key barriers to using EHRs.

**Conclusion:**

EHR was poorly utilized among nurses. Gender, educational qualification, EHR resources, and sponsored training were factors significantly associated with the use. There is an urgent need for comprehensive EHR packages, sustained sponsored training, and formulation of EHR policy for effective EHR implementation.

## INTRODUCTION

Over the years, health records were stored using conventional paper-based records (PBRs) which include data entry forms, admission registers, nursing process records, punch cards, case notes or case files, photographs, and cadets.^1^ Other methods of storage have however evolved, especially with the development of advanced electronic devices, computers, and internet. Advances in EHRs and health information technologies have provided opportunities for storage of health information.^2^ An EHR is the systematized collection of patient and population electronically stored health information in a digital format to improve the quality of healthcare.^3^ An EHR can be described as the digital version of a patient’s paper chart that presents patient’s information promptly and securely to official users. EHRs are among the most promising components of health information technology used to improve the quality and safety of care and at the same time reduce healthcare costs.^4^ In developed nations, it is not uncommon to have patients’ records stored electronically with backups available on various electronic storage devices and media. This is because EHRs’ ability to exchange health information electronically can assist users to provide higher quality and safer care for patients while creating tangible enhancements for the organization. Compared with other data storage methods, EHRs can better increase the quality of care, and guarantee the privacy and security of patients’ data.^5–7^

Despite the well-documented benefits of using EHRs, several implementational barriers have impeded their widespread use. Literature has shown that EHR adoption is at a relatively slow rate by nurses when compared with other developed nations as larger hospitals seem to have been adopting the EHR technologies at an increased rate than smaller hospitals.^4^^,^^8^^,^^9^ PBRs have almost become obsolete in many developed countries^5^ as the countries have advanced in the implementation of electronic nursing documentation systems since EHR has been introduced.^10^ According to the World Health Organization, health information system is 1 of the 6 building blocks for effective health strengthening.^11^ However, only a few healthcare institutions have successfully transitioned into the use of health information storage methods and devices in Nigeria.^1^ There are barriers to the adoption of EHR systems by healthcare facilities in developing countries.^1^^,^^7^^,^^12^ In the study setting, since the introduction of EHR system, there have been various setbacks to the successful implementation of EHRs. The setbacks affect the provision of quality patient care due to several barriers to the adoption of EHRs. The role of nurses cannot be overemphasized in the successful implementation of EHR. Several studies have utilized quantitative research design without an in-depth exploration of factors influencing the utilization of EHRs among nurses.^7^^,^^13^^,^^14^ The quantitative design may not capture the lived experiences and opinions of nurses regarding the use of EHRs. Published data on why nurses in faith-based teaching hospitals prefer PBRs to EHRs in the study setting, in particular, are currently lacking. Hence, this study aimed at adopting a mixed-method design to provide a robust understanding of such data. The findings will equip local policymakers and the institutional stakeholders with necessary information needed to inform policy on sustainable EHRs implementation in the setting.

## METHODS

### Setting

The study was conducted from August to September 2021 in Babcock University Teaching Hospital. It is a mission-based teaching hospital where EHRs have been introduced.

### Study design

A cross-sectional, descriptive, sequential explanatory mixed method was employed in the study.

### Study population

The population included all professional nurses working in Babcock University Teaching Hospital ranging from staff nurses to Chief Nursing Officers.

### Sample size

The sample size was the total study population of 245 nurses currently working in the study setting because the study population was not large, and they all shared similar characteristics. Five nurses out of the total population were not available during the study period. Thus, a total of 240 nurses participated in the survey. Sample size for the qualitative study was done using the principle of “data saturation” whereby the sampling process continued until thematic saturation was reached. During the ninth interview, no new information and no further unique pattern or themes emerged from the participants, indicating data saturation. The researcher conducted 1 more interview after data saturation was evident to ensure that no new information nor further insights emerged, thus further inclusion was stopped. Hence, 10 in-depth interviews (IDIs) sufficiently identified the most prevalent themes.

### Sampling technique

For the quantitative strand, total enumeration sampling techniques were used to enroll all the nurses while purposive sampling of eligible participants across the wards was employed for the qualitative strand.

### Inclusion and exclusion criteria

All registered nurses working in the institution and who gave consent to participate were included in the study. Nurses who were unwell, officially absent from duty, or on leave during the survey were excluded.

### Data collecting tools

A pretested, semistructured, and self-administered questionnaire was used to obtain quantitative data. The findings from previous studies in similar settings^7^^,^^8^ guided the development of the data collection tool. The validity of the tools was ascertained by some electronic health and clinical professionals. The test-retest reliability was measured by Pearson correlation coefficient (*r* = 0.86%). Minor revisions were made to the questionnaire based on the findings of the pilot test. The statistician assisted with the final questionnaire. The questionnaire included different sections, such as sociodemographic characteristics, nurses’ awareness and utilization of EHRs, and factors influencing the utilization of EHRs. For the qualitative strand, data were collected using the tested In-depth inerview (IDI) guide. During the pilot study, a semistructured interview with a nurse was conducted using the IDI guide. There were no major methodological modifications required in the guide. Regarding the qualitative data credibility, the researchers had persistent observation and prolonged engagement with participants during the study. The moderator established a cordial relationship with the participants, to allow them to express themselves freely and easily. The use of peer debriefing method increased the data credibility. The validity strategy for the guide was by member checking and each IDI data was transcribed verbatim and coded accurately to ensure reliability of the data. The principle of data saturation during sampling process was used to ascertain data transferability. Accurate recording of the research procedure over time for easy tracking ensured confirmability of the findings. For findings consistency, all raw information including auditory files, fieldnotes, IDIs questions, and analysis of the results were scrutinized.

### Ethical consideration

Informed consent was obtained and the nature and purpose of the study were explained to the nurses. All participants were assured of confidentiality and anonymity of the information provided.

### Data analysis and management

For the quantitative data, descriptive statistics such as frequency, percentage, mean, and standard deviation were used to summarize and present the results via Statistical Package for the Social Sciences (SPSS version 28). The Chi-square test was used for the bivariate analysis and multivariate analysis was performed using binary logistic regression model. Hosmer and Lemeshow’s goodness-of-fit test was used to assess whether they fulfilled the necessary assumptions. Alpha was set at 5%. Qualitative data were translated and analyzed using a thematic approach with ATLAS.ti version 8. The themes were developed after reflexive reading of the translations. All authors cross-validated emerging themes. IDI questions included are as follows:

1) What are the available resources required for the successful implementation of EHRs in your institution? Probe along the availability of EHR software installed on the computers/mobile phones.
2) What are the enabling and hindering factors influencing the EHR utilization among nurses? Probe various reasons for not using EHRs in their facility.
3) What assistance do the nurses and other healthcare workers need to facilitate the implementation of EHRs in the management of patients in your facility? Probe along if there is any provision of written institutional policy on the use of EHRs.

## RESULTS

### Socio-demographic data of respondents

All the 240 nurses completely and appropriately filled the questionnaires, resulting in a response rate of 100%. The majority of the respondents (47.9%) were younger than 31 years with a mean age of 33.6 ± 8.5. About two-thirds (64.2%) were married, and 72.5% of the total respondents were registered nurses with a degree while 70% had worked in the hospital for 10 years or less (Table 1).

**Table 1.** Socio-demographic data of respondents (*N* = 240)

Variables	Frequency	Percentage
Age (mean + 33.6 ± 8.5)		
<31	115	47.9
31–40	108	45.0
41 and above	17	7.1
Gender		
Male	46	19.2
Female	194	80.8
Marital status		
Single	76	31.7
Married	154	64.2
Divorced	6	2.5
Widowed	4	1.7
Highest educational qualification		
RN and others	66	27.5
BSc./BNSc	152	63.3
MSc/PhD (Nursing).	22	9.2
Years of working		
≤10	168	70.0
11–20	58	24.2
21 and above	14	5.8

### Awareness and utilization of EHRs for health recording

All the nurses in the study were aware of the EHRs with workshops (74.6%) being the major source of their information. Most of the nurses (75.8%) have been trained on EHRs less than 5 years ago (41.7%). Of these, 73.3% of the nurses were institutionally sponsored (Table 2). However, only 24.2% ever used EHRs for documentation of patient health information and services.

**Table 2.** Awareness and training on EHRs (*N* = 240)

Variables	Frequency	Percentage
Ever heard of EHRs		
Yes	240	100
No	0	0
Sources of information		
Workshops	179	74.6
Colleagues	51	21.3
Others	10	4.1
Previous training on EHR		
Yes	182	75.8
No	58	24.2
When trained		
<5 years ago	100	41.7
5 years and above	82	34.2
Never trained	58	24.2
Sponsor of training		
Self-sponsored	6	2.5
Institutional sponsored	176	73.3
Never trained	58	24.2

**Figure 1.**
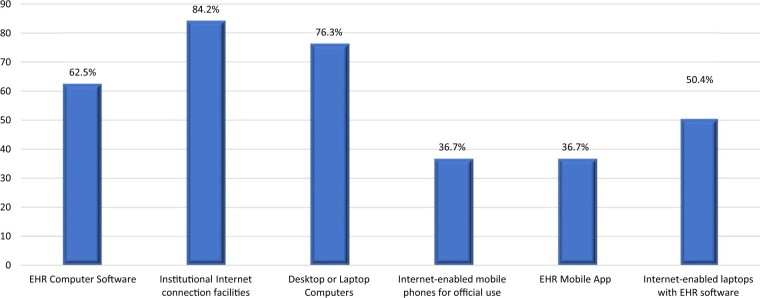
Available electronic solutions for implementation of EHRs.

The respondents reported availability of some electronic solutions in the institution which included EHR computer software (62.5%), institutional internet connection facilities (84.2%), desktops or laptops computers (76.3%), and internet-enabled laptops with EHR software (50.5%). However, very few (36.7%) reported the availability of EHR mobile applications and internet-enabled mobile phones for official use, respectively (Figure 1).

### Factors influencing utilization of EHRs

All the respondents agreed about no provision of incentives or rewards and lack of cooperation by other health professionals as major factors affecting the utilization of electronic records. Moreover, the majority of respondents affirmed that sponsored training (95.8%), encouragement by colleagues and subordinates (90.0%) and poor staff strength (88.8), further influenced the use of EHRs. However, very few respondents (32.9%) regarded ease of use as a factor influencing the utilization of EHRs (Table 3).

**Table 3.** Factors influencing utilization of electronic health records (*N* = 240)

Items	Yes	No
	*N* (%)	*N* (%)
Sponsored training on the use of EHR	230 (95.8)	10 (4.2)
Lack of penalty for not using EHR	183 (76.3)	57 (23.8)
Provision of Incentives or rewards for use of EHR	0	240 (100)
Lack of cooperation by other health professionals	240 (100)	0
Encouragement by colleagues and subordinates	216 (90.0)	24 (10.0)
Ease of use	79 (32.9)	161 (67.1)
High patient workload	158 (65.8)	82 (34.2)
Poor staff strength	213 (88.8)	27 (11.3)

Participants aged 30–39 years had lower odd ratios (OR = 0.35; 95% confidence interval [CI], 0.16–0.76) of utilizing EHRs than those aged less than 30 years. Nurses with BNSc degrees were 4.3 times more likely to utilize EHRs than their counterparts [OR = 4.3; 95% CI, 1.06–17.43]. Nurses with available computer EHR software were 7.4 times more likely to utilize EHRs when compared with their counterparts [OR = 7.4; 95% CI, 0.83–3.81]. Nurses with no previous training on EHRs were 0.8 times [OR = 0.8; 95% CI, 0.02–0.34] less likely to utilize EHRs when compared with their counterparts. Nurses who had their training at least 5 years ago [OR = 1.3; CI, 0.69–2.46] and Sponsored training by the institution [OR = 2.1; CI, 0.24–18.6] had higher ORs of utilizing EHRs (Table 4).

**Table 4.** Bivariate and multivariate logistic regression of factors associated with EHR utilization (*N* = 240)

Variables	Utilization of EHR		OR	95% CI	*P*-value	*χ* ^2^
	Yes (*n* = 48) %	No (*n* = 192%)				
Age					0.093	4.740
<30	35(30.4)	80(69.6)	RC			
30–39	20(18.5)	88(81.5)	0.35	0.16–0.76		
40 and above	3(17.6)	14(82.4)	0.69	0.13–3.51		
Gender					0.002*	9.668
Male	3(6.5)	43(93.5)	RC			
Female	55(28.4)	139(71.6)	1.55	0.21–11.24		
Educational qualification					<0.001*	14.495
RN and others	5(7.6)	61(92.4)	RC			
BSc./BNSc	48(31.6)	104(68.4)	4.3	1.06–17.43		
MSC/PhD (nursing)	5(22.7)	17(77.3)	3.09	0.52–18.47		
Availability of computer EHR software					<0.001*	24.064
No	6(6.7)	84(93.3)	RC	3.04–18.16		
Yes	52(34.7)	98(65.3)	7.429	0.83–3.81		
Availability of EHR mobile App					<0.001*	50.598
Yes	44(50.0)	44(50.0)	RC			
No	14(9.2)	138(90.8)	0.1	0.04–0.21		
EHR training					<0.001*	17.914
Yes	56(30.8)	126(69.2)	RC			
No	2(3.4)	56(96.6)	0.80	0.02–0.34		
When trained					0.001*	18.84
5 years and above	28(34.1)	54(65.9)	RC			
<5 years ago	28(28)	72(72)	1.303	0.69–2.46		
Never trained	2(3.4)	56(96.6)	0.187	0.14–2.45		
Sponsor of training (*n* = 182)					0.001*	18.58
Self-sponsored	1(16.7)	5(83.3)	RC			
Institutional sponsored	55(31.3)	121(68.8)	2.103	0.24–18.6		

Abbreviations: RC: reference category; OR: odds Ratio; χ^2^: chi square.

* Significant *P* < 0.05 at 95% Confidence Interval.

### Qualitative result

Most of the participants were senior nursing officers with at least 5 years of work experience in the institution. The main themes gathered included: availability of resources required for the successful implementation of EHRs, reasons for nonutilization of EHRs in the hospital, and recommendations for EHR implementation. The following subthemes were gathered to buttress the points stressed by the nurses (Table 5).

**Table 5.** Themes and subthemes of EHR utilization

Themes	Subthemes
Available resources for EHR implementation	Functional computer system Constant electricity/solar energy UPS Good internet connection Institutional policy Man power EHR training
Reasons for nonutilization of EHRs	Difficulty to adopt EHRs terminologies Cost implication to run the retraining of staff Workload and inadequate manpower No provision of written policy on the use of EHRs Complexity of the software
Recommendations for EHR implementation	Provision of dependable policies Training of new staff Establishing a follow-up committee

### Available resources required for the successful implementation of EHRs

Participants confirmed the availability of the following resources needed for the successful implementation of EHRs in the institution: functional computer system, constant electricity/solar energy, uninterruptible power supply (UPS), good internet connection or Wi-fi, manpower, and availability of the institutional policy to procedure system. It was further confirmed that the healthcare workers or personnel have been educated on how to go about using EHRs. Computer experts were available to support the training and to guide the users when the EHRs are fully implemented.

Internet and good network coverage is available for effective and lasting implementation of EHR in our facilities [**P3, SNO]**

The individuals who are going to use such records must be educated, know what and what this health record system entails and how to go about using it. That is the first thing, education of the personnel who are going to use the health record. [**P4, SNO]**

Computer system, of course, in every unit. You know a functional computer system. [**P7, SNO]**

#### Installation of EHRs software on computer or mobile tablet

From the findings, participants were not sure if EHRs software has been installed on all the computers or tablets since this has not been implemented by the facility management. Some of the excerpts are as follows:

It’s not yet installed on the computer system. We were just, at a point, trying to train with the hard copy, and let them identify what they are going to see on the system, once they sight it on the paper form, they can know where to go, how to click it on the computer. [**P1, SNO]**

It is likely for EHR to have been installed but I have not operated on the computer. [**P7, SNO]**

Sincerely, I don’t have an idea because the computer has not been switched on in my presence is my inception at the children emergency. [**P6, SNO]**

### Nonutilization of EHRs system in the hospital

Though there have been a series of training on the utilization of EHRs a long-time ago across the units in the hospital, participants noted that EHRs systems have not been implemented by the management. Apart from the long-time training, other factors considered to be barriers to the full operation of and utilization of EHRs in the facility are highlighted below.

#### Difficulty to adopt EHRs terminologies

Participants noted that the implementation stage was so challenging to come up with because it is still a planning stage that requires extensive understanding and adoption of the whole concept.

[…] but the implementation stage was actually the challenge because most of what they came up with in the software are foreign grammar or foreign terminologies; the EHR software did not capture nursing tasks and nursing standardized language. [**P1, SNO]**

#### Cost implication to run the retraining of staff

The management was considering the cost implication to rerun the training for the new health workers again as the majority of the trained health workers (nurses especially) have left the facility or traveled out of the country. Repeating the training will cost a lot of money and the management was unwilling to spend money on how it can be fashioned out. Evidently;

To come up with something new might be a bit challenging because it will be a drawback for the new nurses to blend into, except we have to train again, and training is going to cost us a lot of money. These are the challenges, the cost implication and all that might be a drawback. [**P1, SNO]**

#### Workload and inadequate manpower

The report showed that nurse-to-patient ratio was low. There were many patients to a few nurses, and this consumed a lot of time in attending to patients in the ward. There was no sufficient number of nurses to work as a result of the brain drain. Also, institutions keep having additional units that invariably need the services of adequate manpower to maintain standard and for the effective management of records.

Yes, patients’ workload may be an issue. If you have too many patients but that depends on emm…how fast an individual can provide data because if you are not so learned about the use of computer you might, it might limit you from attending to much patients. [**P5, SNO]**

Now we have a neurosurgeon and in the statistics in the last 5 -6 months, he has the highest number of patients, which was not like that before. Thus, we have more patients, and for that we might need an additional man –power, additional nurses to manage his patients. [**P1, SNO]**

[…] staff brain drain affects the operation of EHR, as some of those that were trained have left the country for greener pasture. It will require management to retrain new staff again. Which is a whole lot of financial implication on the side of management. [**P4, SNO]**

#### No provision of written policy on the use of EHRs

It was noted by the participants that the institution has not come up with a better policy regarding the implementation of EHRs.

No. No policy, probably because some staff have issues then and could not be trained on EHR, the management has not written any policy on utilizing the EHRs. [**P4, SNO]**

[…] the challenge currently is from the management that is the institution with the commencement policy, because once something is not commencing, people won't comply[…]. [**P5, SNO]**

#### Complexity of the software

One of the reasons for the nonimplementation and utilization of EHRs was the complexity of the software for the users. A participant was of the opinion that the modality of software to be used is complex and not friendly to adopt.

[…] but in terms of the software, I think that is where we have the issues. There is a problem with the connection, that is interconnectivity of the various umm…umm… umm…interdisciplinary teams, I mean the various disciplines in the health care system. So maybe that is what also delaying this because if it is about the hardware the computer system and CPU and whatever they are, all are readily available to commence and there is adequate knowledge of the personnel who are to use it, the personnel have already been educated on using it. The hardware to commence is available…

#### Recommendations for implementation and utilization of EHRs

Participants recommended that: the institution should provide dependable policies that people can adapt to; bring more expertise to educate more staff, new staff should be opened to more training, and establish a follow-up committee that will monitor compliance with the usage. In addition, the hospital can put the assignment in the hands of computer experts of the university to make it easy for its smooth running.

They will inform all health professionals including the pharmacists, radiologists, also scientists, Health Information Management, nurses, and medical doctors. So it should go round aside from them making policy, if they make policy and those people that supposed to be aware of the policy are not aware, it won’t be effective. [**P2, SNO]**

Yes, a good emm… developer of software. They can take it up as a project if given to their students. They could handle it, surprisingly you might even get better softwares which would be better than what we've been going about and struggling with. That is one of my suggestions. **[P2, SNO]**

…the hospital management should be approached and educated about EHRs. The management needs to be enlightened on benefits of using EHRs compared to paper and pen. I believe if they (institutional authorities) are properly informed about the benefits, it will hasten the implementation of the EHRs. **[P8, PNO]**

## DISCUSSIONS

An EHR system is one of the important tools for achieving and optimizing the delivery of quality health care as well as means for clinical data for biomedical research.^15^ In the research setting, the process of implementing a functional EHR has been in the pipeline for almost a decade, yet various factors mitigate against the prompt implementation of EHRs. It becomes imperative to assess the utilization of EHRs among nurses and associated factors in the study setting; implementation in the nearest future may be hinged on the findings from this study.

Studies have shown similar findings to our studies that most of the nurses were females and had bachelor’s degree qualifications.^7^^,^^10^^,^^15^^,^^16^ In contrast to other studies however,^7^^,^^17^ the current study showed that a higher proportion of nurses had received EHR training sponsored by the institution, had a wide availability of institution-owned electronic solutions, and an enabling environment. In contrast, previous studies showed that nurses were willing to use EHR system but lacked training and necessary technological devices.^7^^,^^18^ Thus, lack of proper education and training on EHR can threaten the utilization of EHRs and negatively impact the quality of nursing documentation.

However, despite the respondents’ adequate training on EHR, only a few utilized EHRs in recording patient’s care in the setting. Similarly, a study on factors influencing the adoption of EHRs in public health facilities in Kenya revealed that the potentials of EHR system were yet to be maximally attained as it was poorly (11.1%) utilized in the settings.^19^ Moreover, Habibi-Koolaee et al^16^ reported that despite half of the nurses using computers at home (50.2%), only 32% used computers at work. In a recent study, a poor level of utilization of EHR for storage of patient’s health information was noted in public hospitals in Nigeria.^7^ Contrary to our findings, Ademuyiwa et al^20^ found from a study on knowledge and use of Nursing Informatics among Nurses in a University teaching hospital in Nigeria, that almost all (96%) of the healthcare workers had ever used health informatics. The discrepancy may be due to the fact that the university setting is larger than this study setting. Lower EHR usability was associated with higher odds of burnout, job dissatisfaction, and intention to leave when compared with nurses working in hospitals with better EHR usability.^21^ Therefore, identifying factors influencing the use of EHRs will help to devise strategies to enhance the use of EHRs by nurses and other health disciplines in the study setting.

This study, in contrast to the findings in Kenya,^19^ further revealed that respondents with functional EHR-related infrastructure such as computer EHR software and sponsored EHR training had higher likelihood of utilizing EHRs when compared with their counterparts. Excerpts from the IDIs also corroborated the fact that stable internet connectivity and power supply were available for the effective operation of EHR in the facility. However, the current brain drain among Nigerian nurses has increased the workload which negatively impacts the effective utilization of EHR in the facility. Respondents reported that most of the nurses who were recently trained have relocated while those who had training a long-time ago and the newly recruited nurses who were never trained were less likely to use EHRs. Other previous findings have supported our findings that supportive training on EHR in health organizations will enhance healthcare workers’ use of EHRs and less resistance to stopping EHR usage.^15^^,^^22^^,^^23^ Therefore, all nurses should not only have EHR training but also have periodic and sponsored technical training on EHR systems to enhance compliance with the use of EHR in order to promote patient care. There should also be refresher courses on EHR operations among nurses and other health workers.

Moreover, this study also showed that there was a significant association between respondents’ socio-demographics and utilization of EHRs as found in other studies. Gender, age, and educational level were the important factors that had positive impact on the utilization of EHR. This depicts that nurses with higher educational qualifications tend to easily adopt the use of technologies because their baccalaureate programs tend to include more computer-based learning modalities than diploma programs. Also, females and younger generations are now using technologies and social media for a large part of the day to exchange information. Thus, they may have an easier time to adapt with the technology-oriented changes in their institution.

Nurses however perceived that collaboration and cooperation among various medical specialties is a key factor in achieving the implementation of EHR in patient care. Muhaise et al^12^ noted that one of the factors that can influence the adoption of EHR was the need for involvement and participation of all stakeholders in the health sector, and a good change management strategy and leaders. Other factors influencing the implementation of EHR in the study setting included lack of enforcement of penalty for not using EHR and provisions of certain incentives for those that comply with the use of EHR in the institution. It is believed that monetary incentives may serve as an external motivation especially to nurses having high records of EHR utilization, thus propelling them to adopt and continually use EHR. This finding also supported the policy of federal government in providing required funds necessary for compliance with the meaningful involvement in the EHR system among healthcare practitioners.^23^ Similarly, a qualitative study assessing factors to overcome barriers affecting electronic medical record usage by physicians revealed that giving monetary incentives can overcome barriers to using EHR by physicians.^14^ Staff incentive programs such as rewards and benefits are therefore needed to be designed to attract, engage, and retain staff to feel more motivated in doing their best to continuously utilize EHRs.

The current study also showed that a significant number of the nurses affirmed ease of use of EHR systems as a factor influencing its use. This could be attributed to the fact that not all nurses in the study setting were exposed to EHR training. Pugal et al^18^ noted in their study that most nurses lacked competence and confidence in the use of EHR due to the limited number of EHR training they had. However, other studies in Nigeria,^7^^,^^13^ and Jordan,^10^ revealed that most nurses demonstrated the usefulness and ease of use of EHRs. EHR’s difficulty-to-use was reported as one of the important barriers to EHR use.^14^ A qualitative study on user perspectives on barriers to the implementation of EHR in Bangladesh^4^ revealed low levels of computer proficiency among nurses, complexity of the system, and legacy systems resistance. A narration from this study supported the findings from the study in Bangladesh.^4^

[…] implementation stage was actually the challenge because most of what they came up with in the software are foreign grammar or foreign terminologies and the EHR software did not capture nursing tasks and nursing standardized language. [**P1, SNO]**

Hence, nurses need to update their professional and technological knowledge to keep abreast with the current EHR standards and to improve their confidence level. The hospital management should ensure regulations for EHR system interoperability. The participants suggested that EHR developer should incorporate some of the nursing tasks and standardized languages into the EHRs software for more nurses to embrace its use. The developer should also engage nurses throughout the stages of implementation to identify and resolve problems that users might encounter while using the software. Some participants also narrated that harmonized standard legal enforcement and policy framework on EHR utilization by the hospital management are currently lacking. Lack of legal enforcement of EHR was equally reported as a factor contributing to low utilization of EHR in some hospitals.^19^ It is therefore recommended that the hospital should take proactive steps to identify and harmonize legal policy structure to implement EHR for effective healthcare service delivery.

## LIMITATIONS

The target population included all the nurses working in a tertiary healthcare institution with facilities for training, research, and quality service delivery. Involving all other healthcare workers and key informants dealing with information relating to patients’ care may provide more information about some other factors that may influence the implementation and utilization of EHRs. However, the use of self-reported questionnaires and one-on-one interviews used for data collection from the respondents prevented making biased inferences from responses, thus the strength of this study.

## CONCLUSION

The proportion of nurses currently using EHRs was low. Factors associated with the utilization of EHRs included gender, educational qualification, EHR resources, and sponsored training. Difficulty to adopt EHRs terminologies, cost implication to run the retraining of staff, excess workload, inadequate manpower, and lack of written policy on the use of EHRs were the main identified themes for the key barriers to using EHRs. There is urgent need for the provision of comprehensive EHR packages, sustained sponsored training of staff, and formulation of EHR policy in this setting for effective EHR implementation.

## Supplementary Material

ooad059_Supplementary_Data

## Data Availability

The data underlying this article are available at *JAMIA Open* online.
